# Erbium Luminescence Centres in Single- and Nano-Crystalline Diamond—Effects of Ion Implantation Fluence and Thermal Annealing

**DOI:** 10.3390/mi9070316

**Published:** 2018-06-22

**Authors:** Jakub Cajzl, Pavla Nekvindová, Anna Macková, Petr Malinský, Jiří Oswald, Zdeněk Remeš, Marián Varga, Alexander Kromka, Banu Akhetova, Roman Böttger, Václav Prajzler

**Affiliations:** 1 Department of Inorganic Chemistry, University of Chemistry and Technology, Technická 5, 166 28 Prague, Czech Republic; pavla.nekvindova@vscht.cz (P.N.); akhetova@vscht.cz (B.A.); 2 Nuclear Physics Institute, Academy of Sciences of the Czech Republic, v.v.i., 250 68 Řež, Czech Republic; mackova@ujf.cas.cz (A.M.); malinsky@ujf.cas.cz (P.M.); 3 Department of Physics, J. E. Purkinje University, České Mládeže 8, 400 96 Ústí nad Labem, Czech Republic; 4 Institute of Physics, Czech Academy of Sciences, v.v.i., Cukrovarnická 10/112, 162 00 Prague, Czech Republic; oswald@fzu.cz (J.O.); remes@fzu.cz (Z.R.); varga@fzu.cz (M.V.); kromka@fzu.cz (A.K.); 5 Institute of Ion Beam Physics and Materials Research, Helmholtz Zentrum Dresden-Rossendorf, 01328 Dresden, Germany; r.boettger@hzdr.de; 6 Department of Microelectronics, Faculty of Electrical Engineering, Czech Technical University in Prague, Technická 2, 166 27 Prague 6, Czech Republic; vaclav.prajzler@fel.cvut.cz

**Keywords:** nano-crystalline diamond, erbium, ion implantation, luminescence, rutherford backscattering spectrometry (RBS), Raman spectroscopy, thin films

## Abstract

We present a fundamental study of the erbium luminescence centres in single- and nano-crystalline (NCD) diamonds. Both diamond forms were doped with Er using ion implantation with the energy of 190 keV at fluences up to 5 × 10^15^ ions·cm^−2^, followed by annealing at controllable temperature in Ar atmosphere or vacuum to enhance the near infrared photoluminescence. The Rutherford Backscattering Spectrometry showed that Er concentration maximum determined for NCD films is slightly shifted to the depth with respect to the Stopping and Range of Ions in Matter simulation. The number of the displaced atoms per depth slightly increased with the fluence, but in fact the maximum reached the fully disordered target even in the lowest ion fluence used. The post-implantation annealing at 800 °C in vacuum had a further beneficial effect on erbium luminescence intensity at around 1.5 μm, especially for the Er-doped NCD films, which contain a higher amount of grain boundaries than single-crystalline diamond.

## 1. Introduction

Nanocrystalline diamond (NCD) is a material that retains outstanding properties of single-crystalline diamond [1,2]. It also has high refractive index, low optical absorption scattering (especially in near-infrared region), the highest hardness and thermal conductivity, and it is also biocompatible. The photoluminescence related to diamond colour centres such as, e.g., Si, N, Ni, C, Cr is nowadays attracting growing attention owing to its highly promising applications in photonics, spintronic, quantum information processing and nanomedicine [3,4,5,6,7,8,9,10,11]. So far, the diamond colour centres have mostly been identified as single-photon emitters with emission wavelengths shorter than approx. 900 nm [12]. The substitutional rare-earth impurities extend the palette of colour defects in the infrared region with useful magnetic or optical properties, leading, for instance, to the possibility of applications as light-emitting devices. 

The shielding of the internal 4*f* orbitals by external 5*s* and 5*p* orbitals makes the lanthanides very narrow-line and stable luminescent centres [13]. Erbium, due to its strong narrow emission at a wavelength of about 1530 nm, is one of the most frequently used lanthanides in photonics [14,15,16]. The Er luminescence in the crystals can be affected by: (i) erbium oxidation state (+III is a stable configuration with intensive emission); (ii) erbium concentration (higher concentration yields higher luminescence intensity, too high concentration—usually above 6.0 at. % [17]—however, causes luminescence quenching); (iii) a crystal field in the vicinity of erbium ions (the effect on the probability of energy transitions of Er) [18,19].

Two main techniques are generally used for the diamond doping: (i) ex-situ ion implantation and (ii) in situ doping during the chemical vapour deposition (CVD). Ion implantation concerns, e.g., incorporation of N, Si, Ni, Cr, C, Ce, Gd, He atoms, etc., at various implantation fluences up to 1 × 10^17^ ions·cm^−2^) as well as energies (30 keV–2.5 MeV) depending on the implanted elements [20,21,22,23,24]. Due to the damaging of diamond lattice by ion implantation, thermal annealing at high temperature (hundreds of °C) should be performed to recover or reorganize the crystal lattice. However, it has been known that in spite of oxide materials, recovery of the diamond structure is quite difficult as it tends to graphitize [25,26]. Analysis of the diamond structure before and after ion implantation, as well as the subsequent annealing process, is commonly done by Raman spectroscopy [27].

Apart from the chemical nature characterization, the investigation of the implanted elements luminescence activity and their efficiency is also important. Concerning the development of photonics and quantum-based networks operating with commercial optical fibres operating in the near-infrared (NIR) region of about 900–2000 nm, it is particularly interesting to study an efficient incorporation of Er into the diamond [28]. According to the theoretical modelling results, which were done in [11] as well as in our previous work [29], the lantahanide ions (Er, Eu, etc.) are able to exist both in substitutional and interstitial positions in diamond without serious parent-diamond structure changes or bond cracking. Comparing the cohesive energies of the different structural models, a more probable erbium location in diamond is the substitutional site of carbon with one or three vacancies in the close vicinity of erbium atom. In both works low room-temperature photoluminescence (of erbium or europium) in diamond was detected. 

This paper builds on the previous theoretical calculations and experiments and focuses on the erbium photoluminescence enhancement in Er-doped single- and nano-crystalline diamond. In addition, the software package Stopping and Range of Ions in Matter (SRIM) [30] is used for the Monte Carlo (MC) simulations of the defect depth distribution in pure diamond materials and/or diamond-like films formed after the Er implantation [31]. Rutherford Backscattering Spectrometry (RBS) is used to determine the Er concentration depth profiles in the prepared samples. The diamond structure damaging (caused by ion implantation) and post-implantation recovery (annealing) is investigated by Raman spectroscopy. 

## 2. Materials and Methods 

### 2.1. Samples Preparation

Pure silica glass (10 mm × 10 mm × 1 mm) was used as the substrate for NCD thin film deposition from the CH_4_/CO_2_/H_2_ gas mixture using a pulsed linear-antenna microwave plasma CVD process (modified system AK 400, Roth and Rau, AG, Hohenstein-Ernstthal, Germany). The diamond-growth conditions were as follows: 2.5% of CH_4_ and 10% of CO_2_ as compared with hydrogen, pulsed microwave power of 2 × 2 kW, the total gas pressure of 10 Pa, the deposition time of 30 h and the substrate temperature of 650 °C. The thickness of the diamond films was about 280 nm and grain size was up to 300 nm. The reference Er-doped diamond samples were prepared from single-crystalline diamond wafers (3 mm × 3 mm × 0.3 mm) with the <001> crystallographic orientation (purchased from Element Six Technologies Ltd., London, UK). Both substrate types were exposed to Er implantation at three different ion implantation fluences (1.0 × 10^14^, 1.0 × 10^15^ and 5.0 × 10^15^ cm^−2^) with 190 keV Er^+^ ions. The implantation energy of 190 keV was chosen to create the sub-surface layer of erbium-doped diamond structure.

Finally, the Er-doped diamond samples were annealed for 1 h either in Ar atmosphere or in vacuum (10^−4^ mbar) at three different temperatures (400, 600 and 800 °C). 

### 2.2. Samples Characterization

The effect of the different ion implantation fluences and thermal annealing temperatures on thin films was analysed by Raman spectroscopy. Raman analysis was performed on a Thermo Scientific DXR Raman Microscope spectrometer (Thermo Fisher Scientific, Waltham, MA, USA) equipped with a confocal Olympus microscope (Olympus Corporation, Tokyo, Japan). The excitation source used was a solid-state Nd:YAG laser (wavelength: 532 nm, maximum power: 10 mW). The Raman measurements were acquired at power of 2–8 mW, 10 accumulations of 10 s scans, grating with 900 lines/mm and a 25-µm pinhole aperture. As a detector, a multichannel thermoelectrically cooled charge-coupled device (CCD) camera was used. A magnification of 50× provided a measurement spot-size of ~1 µm^2^. The peak-fitting (deconvolution) process was used to determine the spectral components as well as the peak positions, widths and intensities. The deconvolution fitting of Raman spectra was carried out in the range of 920–1800 cm^−1^ using Voight, Gaussian and Lorentzian functions according to the parameters given in Table 1. Prior to the fitting process, a linear background subtraction was done. According to [32,33], the ratios of the characteristic main peak/band intensities and areas were evaluated to obtain information on the diamond-structure changes.

The Er concentration depth profiles in NCD films were measured by rutherford backscattering spectrometry. The analysis was performed at a Tandetron 4130 MC accelerator (Nuclear Physics Institute, v.v.i., ASCR, Řež, Czech Republic) using a 2.0 MeV He^+^ ion beam. RBS-channelling analysis served for the structural study of the erbium implanted single-crystalline diamond. The depth profiles of displaced atoms were deduced from the RBS-channelling aligned spectra of Er-implanted single-crystalline samples using a beam of 1.7 MeV He^+^ ions from a Tandetron accelerator. The collected data were evaluated and transformed into depth concentration profiles using SIMNRA 6.06 [34] codes, utilising cross-section data from IBANDL [35].

**Figure 1:**
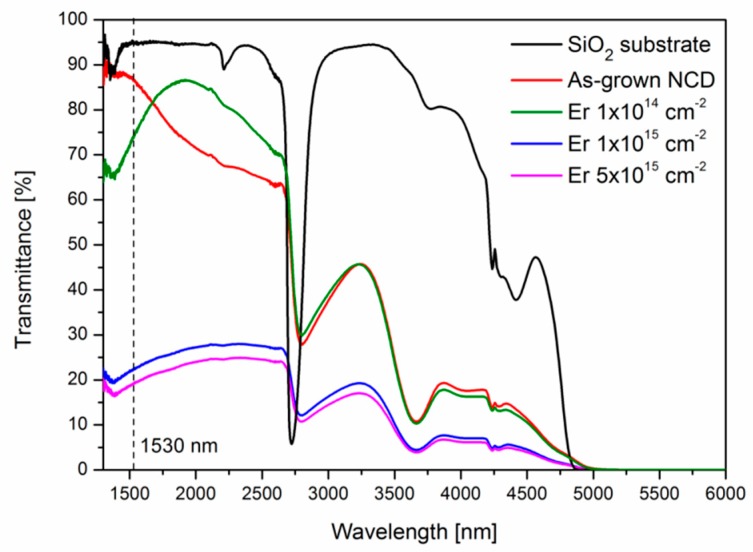
NIR transmittance spectra of the SiO_2_ substrate, as-grown nano-crystalline (NCD) and the prepared samples (after Er ion implantation and annealing at 800 °C). All the transmittance percentage values are relative values according to the background.

The prepared samples were characterized by NIR transmittance spectroscopy using ThermoFisher Scientific Nicolet iS5 in the range of 1300–5000 nm. The spectra of NIR transmittance of the SiO_2_ substrate, as-grown NCD and the prepared samples (after Er ion implantation and annealing at 800 °C) are depicted in Figure 1. All the samples (as-grown, implanted and annealed) are transparent in the NIR region. From the spectra, it is possible to evaluate that the as-grown NCD had a transmittance of around 87%. After the Er ion implantation using the lowest implantation fluence of 1.0 × 10^14^ cm^−2^ and annealing at 800 °C, the transmittance slightly decreased to around 74%. Using the two highest Er implantation fluences of 1.0 × 10^15^ and 5.0 × 10^15^ cm^−2^ with annealing at 800 °C caused substantial decrease of transmittance to around 23% and 19%, respectively. 

The photoluminescence spectra of the investigated samples were collected at room temperature in the range of 1440–1650 nm. A semiconductor laser POL 4300 emitting at 980 nm was used for the excitation. The luminescence radiation was detected by a two-step-cooled Ge detector J16 (Teledyne Judson Technologies, Montgomeryville, PA, USA). To scoop specific wavelengths, a double monochromator SDL-1 (LOMO, Saint Petersburg, Russia) was used. 

## 3. Results 

### 3.1. Er-Induced Defects Depth Distribution—Simulations Using SRIM

**Figure 2:**
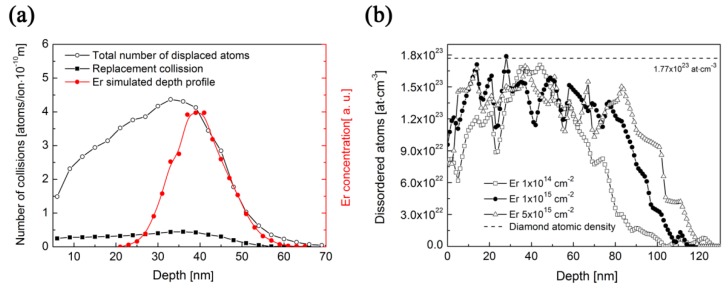
(**a**) Stopping and Range of Ions in Matter (SRIM)-simulated Er concentration depth profile in a pure diamond structure with a density of 3.5 g·cm^−3^ and the depth profile of displaced atoms produced in a diamond structure as simulated by SRIM; (**b**) Theoretically determined atomic density depth profile of displaced atoms for different implantation fluences using experimentally-determined channelling spectra of erbium in single-crystalline diamond with the same energy [29].

For a better understanding of the ion implantation-induced damage in the implanted films, the software package Stopping and Range of Ions in Matter (SRIM) was applied for the simulation of the defect depth distribution. SRIM is a well-established simulation code including the Monte Carlo (MC) simulation of all ion/target-atom collisions that lead to the target damage simultaneously with the simulation of the implanted-ion depth. The deceleration of ion beam is accompanied by electronic and nuclear stopping, the interplay of which is crucial for the structural changes of irradiated material. Electronic stopping generally leads to atom ionisation and excitation, and afterwards to the creation of free radicals and free chemical bonds. The nuclear stopping leads to atomic displacement and the production of vacancies or large-scale defects [30]. The Transport of Ions in Matter (TRIM) Monte Carlo code of the SRIM package simulates the depth profiles of irradiation-induced vacancy-interstitial Frenkel pairs (FP) using the binary-collision approximation method. Under the assumption that each FP arises in a single atomic displacement, it is possible to simulate the vacancy profile (see Figure 2a, where the total vacancy depth profile generated by Er ions in a diamond layer is simulated simultaneously with the Er-implanted depth profile). 

In order to have experimental data on the displaced atoms created by Er ion implantation, the single-crystalline diamond was implanted together with NCD films where the RBS channelling was employed to obtain the displaced-atom depth profiles from the channelling spectra in the single-crystalline diamond. The yields of the RBS-channelling aligned spectrum, at a selected depth *z*, increase by the direct scattering of the channelled component from displacements and the scattering of the de-channelled component from lattice atoms. The use of the known minimum-yield depth profiles χ_D_ (*z*), which are deduced from the RBS-aligned spectra, makes it possible to extract the depth profiles of the displaced atoms by iterating the aligned spectrum yield and converting it into the dislocated-atom density using the approach described in [36]. The RBS channelling spectra of the single-crystalline diamond implanted by Er can be found in [29], and the extracted displacement atom concentration depth profile is presented in Figure 2b. The production of the displaced atoms is supposed to be similar for both single- and nano-crystalline diamond structures. It is clear from Figure 2b that the density of the total number of displaced atoms exhibits the concentration maximum in the depth of about 40 nm, which is appropriate to the projected range of erbium ions. The number of the displaced atoms per depth slightly increases with the fluence, but in fact the maximum reaches the fully disordered target even in the lowest ion fluence used. The experimentally determined defect depth profiles for the particular fluences reveal the progressive defect of layer thickness enhancement with ion implantation fluence, where the displaced atoms are observed in the depth of about 120 nm for the highest ion implantation fluence used (see Figure 2b). One can see that the structure is entirely damaged within the projected range of erbium (the depth of about 40 nm). The SRIM-predicted Er concentration depth profiles for erbium ions implanted with an energy of 190 keV to a single-crystalline diamond are presented in Figure 2a. The projected range *R_p_* is of about 40 nm and the standard deviation Δ*R_p_* is about 6 nm. However, the RBS channelling results show that the fluence of 1.0 × 10^14^ cm^−2^ causes a lower thickness of damaged layer than higher fluences. 

The depth distribution of all displaced atoms simulated by SRIM—the sum of vacancies and replacement collisions—and the replacement collisions are also shown in Figure 2a. Experimentally determined atom displacement depth profiles in Figure 2b are in a rough agreement with the SRIM simulation presented in Figure 2a for the lowest ion implantation fluence. Higher ion implantation fluences caused a gradual damage build-up in the implanted layers leading to a broadening of the damaged layers.

### 3.2. Er-Ion Implantation—As-Implanted Samples Characterization

**Figure 3:**
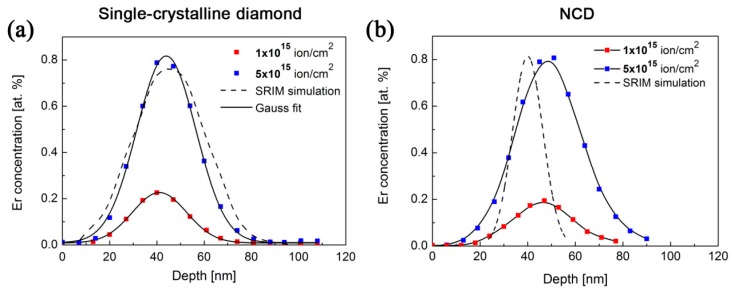
Erbium concentration depth profiles for various implantation fluences determined by the RBS method in (**a**) single-crystalline diamond and (**b**) nano-crystalline diamond thin films.

The RBS method was used to determine Er concentration depth profiles in the prepared single- as well as nano-crystalline diamonds. Figure 3 shows the Er concentration depth profiles of the as-implanted samples as a function of ion implantation fluences along with the SRIM simulation-predicted Er depth distribution profile. 

The concentration profile for a fluence of 1 × 10^14^ cm^−2^ is limited by the background noise in the RBS spectrum and is not shown in Figure 3. The Er concentration depth maximum determined for NCD films is slightly shifted to the higher depth with respect to the SRIM simulation (SRIM simulation was taking into account the single-crystalline diamond density of 3.5 g·cm^−3^). The Δ*R_P_* values are slightly higher for NCD films than for single-crystalline diamond. Such a difference is attributed to the presence of the grain boundaries in NCD; i.e., the different density of the present *sp*^2^ and *sp*^3^ phases. The structural differences between NCD films and single-crystalline diamond, which are not included in SRIM simulation, cannot be taken into account in RBS analysis. The observed values for the Er concentration depth profiles parameters (the values of the maximum concentration depth *R_P_* and the standard deviation Δ*R_P_*) for NCD films and single-crystalline diamond implanted with Er under the same implantation conditions are summarized in Table 2.

**Figure 4:**
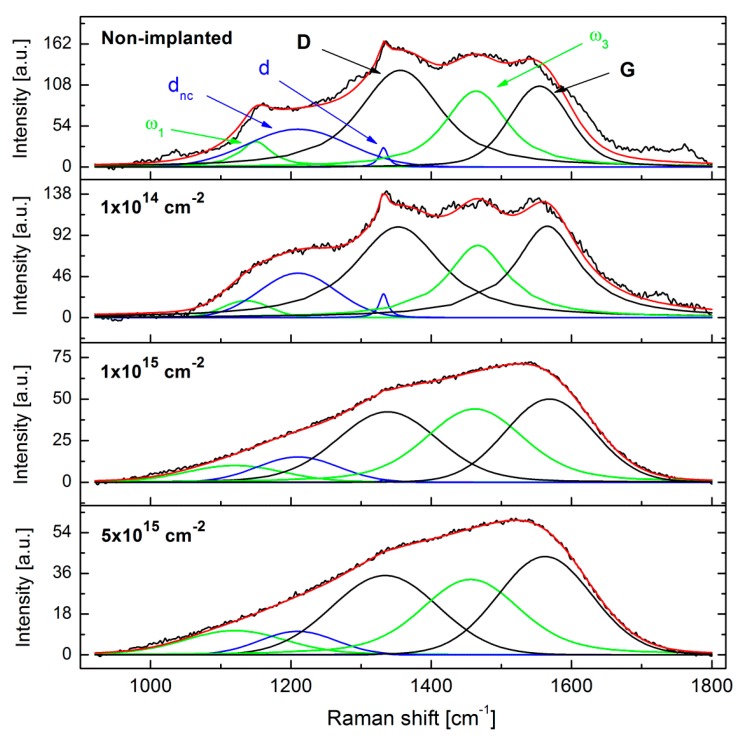
The Raman spectra of NCD films implanted with Er atoms using various implantation fluences—for the comparison also the non-implanted sample is shown. The measured spectra are marked by the black curve, the fitted spectra by the red curve. Various components of the peak-fitting (deconvolution) procedure are depicted using dissimilar colours and labelled in the Raman spectrum of the non-implanted NCD film.

Figure 4 shows the as-measured and deconvolution-fitted Raman spectra of the NCD films implanted using different erbium ion implantation fluences. The Raman spectrum of NCD consists of six main characteristic peaks: (i) the sharp line related to the single-crystalline diamond (*sp*^3^ carbon hybridisation in the crystalline state) observed at 1332 cm^−1^ (labelled as the d-peak); (ii) the graphite (*sp*^2^ carbon hybridisation in the crystalline state) band at ~1580 cm^−1^ (labelled as the G-band); (iii) the shoulder at 1350 cm^−1^ (labelled as the D-band) assigned to the breathing modes of carbon with *sp*^2^ hybridisation in rings; (iv) the two peaks at 1150 cm^−1^ (ω_1_) and 1480 cm^−1^ (ω_3_) assigned to trans-polyacetylene (t-PA) on the grain boundaries denoted as ω_1_ and ω_3_, respectively; and (v) the band around 1200–1250 cm^−1^ (denoted as d_nc_), which corresponds to the broadened vibrational density of states (VDOS) of small diamond clusters and/or tetrahedral amorphous carbon (denoted as ta-C and having *sp*^3^ hybridisation) [33,37,38,39].

For the lowest ion implantation fluence (1.0 × 10^14^ cm^−2^), the Raman spectrum is similar to the spectrum of the non-implanted sample; however, some minor differences can be found after the deconvolution analysis. Ion implantation fluences higher than 1.0 × 10^14^ cm^−2^ already resulted in a significant degradation of NCD films presented by the disappearance of the diamond peak at around 1332 cm^−1^. According to [27], significant bands in the measured Raman spectra can be assigned to the amorphous carbon in tetrahedral coordination (*sp*^3^ hybridisation) together with glassy carbon (*sp*^2^ hybridisation). This implies that the Er implantation fluences greater than 1.0 × 10^14^ cm^−2^ lead to a significant graphitisation (and/or the conversion of hybridisation from *sp*^3^ to *sp*^2^) of the diamond layer. 

**Figure 5:**
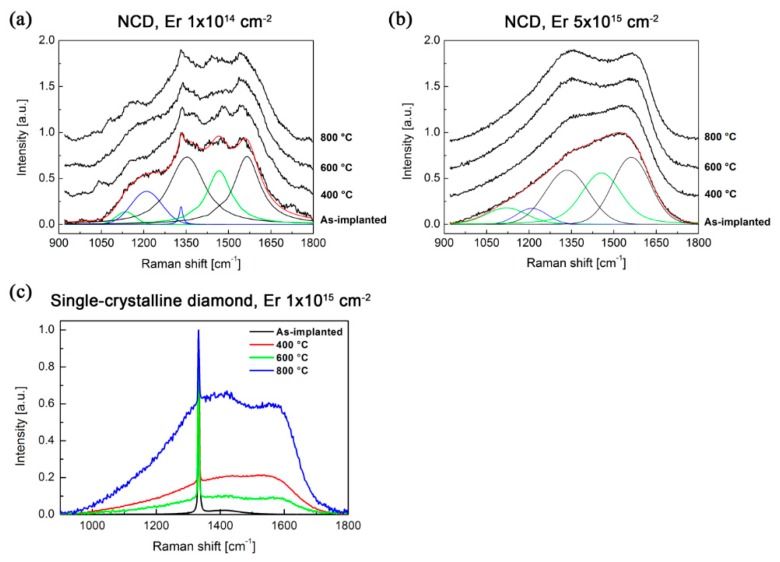
The Raman spectra of the single- and nano-crystalline diamond samples implanted with various fluences and annealed at temperatures ranging from 400 °C to 800 °C. The deconvolution analysis of NCD samples is shown for the spectra of the as-implanted NCD sample (the description of the particular deconvolution bands is explained in Figure 4). The single-crystalline sample is normalized to (0, 1) for better comparison.

The Raman spectrum of the single-crystalline diamond sample implanted with the implantation fluence of 1.0 × 10^15^ cm^−2^ with characteristic peak centred at 1332 cm^−1^ is shown in Figure 5. The diamond peak is related to the *sp*^3^ hybridised carbon (the same carbon hybridisation as in the diamond structure), not in the amorphous phase, but rather in the crystalline phase. The observed broad band at approx. 1400–1450 cm^−1^ can be attributed to the damaged sub-surface layer of implanted diamond samples i.e., can represent either trans-polyacetylene chains at diamond grain boundaries or disordered *sp*^2^ bonded carbon within the damaged diamond sub-surface [40].

Measurement of the luminescence of as-implanted samples showed (for both single- and nano-crystalline samples) that the intensity of luminescence at 1530 nm is very low and hardly resolvable in the background signal (noise).

### 3.3. Post-Implantation Thermal Annealing—As-Annealed Samples Characterization

To recover the diamond crystal structure and to enhance the Er-related photoluminescence, the thermal annealing in Ar atmosphere, as well as in vacuum, at three different temperatures (400, 600 and 800 °C) was employed for all the samples. The Raman spectra were measured and evaluated for each annealing temperature. Since argon with a purity of 99.996% was used, the results showed that annealing in argon atmosphere caused a serious degradation of the surface layer, and therefore was not written about and discussed in the text. Due to the trace amount of oxygen, annealing in Ar atmosphere (especially at higher temperatures of more than 600 °C) did not lead to NCD structure recovery—the Raman spectra had very low intensities with hardly resolvable diamond peaks/bands. On the contrary, thermal annealing in vacuum caused a partial recovery of the diamond structure. Thus, the effect of annealing in vacuum has been studied more in detail for both single- and nano-crystalline samples. 

For the determination of the structure changes in Er-doped NCD we employed the spectra peak deconvolution procedure [33,37,38,39]. The data evaluated from the deconvoluted Raman spectra of the Er-doped NCD samples, i.e., the intensity ratios (I_D_/I_G_ and I_d_/I(d_nc_)) and area ratios (A_D_/A_G_), are summarised in Table 3. In the table, the calculated amounts of *sp*^3^ hybridised carbon atoms (from G-band positions as well as from A_D_/A_G_ ratios) correspond to the amount of tetrahedral amorphous carbon with *sp*^3^ hybridisation—not the crystalline diamond *sp*^3^ phase [32]. Therefore, these values refer to the amorphous carbon on grain boundaries as well as inside grains (where after the ion implantation and annealing can also be some small amount of amorphous carbon phase). The corresponding Raman spectra of NCD samples are shown in Figure 5a,b. Figure 5c shows the effect of the annealing on the Raman spectra in the single-crystalline substrate implanted using fluence 1 × 10^15^ ions.cm^−2^ and annealed in vacuum at the same conditions as NCD samples. 

The I_d_/I(d_nc_) ratio shows the amount of “nanocrystallinity” of the diamond films [33], whereas the G-band position identifies the amount of *sp*^3^-coordinated carbon atoms in amorphous phase (i.e., carbon atoms on the grain boundaries in contrast to the crystalline in-grain *sp*^3^ hybridised carbon atoms indicated by *d* peak). We observed that the Raman spectrum of the NCD sample implanted at the lowest implantation fluence (1 × 10^14^ cm^−2^) substantially differs from the samples implanted at higher implantation fluences (1 × 10^15^ and 5 × 10^15^ cm^−2^)—see Figure 5a,b. For the lowest fluence of 1 × 10^14^ cm^−2^ (i.e., below the amorphisation limit, see Section 3.2), the values of I_d_/I(d_nc_) ratio and the amount *sp*^3^ hybridised carbon atoms in the amorphous phase were very similar to those of the non-implanted NCD sample. Post-implantation annealing of up to 600 °C did not result in significant changes to the Raman spectra. In contrast, for the temperature of 800 °C and the fluence of 1 × 10^14^ cm^−2^ increase in the I_d_/I(d_nc_) ratio from around 0.5 to 0.9 was observed. This indicates a partial recrystallisation of the in-grain diamond structure, resulting in a higher amount of the diamond phase (*sp*^3^ hybridised carbon atoms in crystalline phase) and a more pronounced diamond peak in the Raman spectrum. In this sense, the annealing in vacuum at the temperature of 800 °C (and presumably higher, too) is required to partially recover the NCD structure. 

For fluences of 1 × 10^15^ and 5 × 10^15^ cm^−2^ samples had almost identical spectra after implantation as well as after the annealing at different temperatures. Figure 5 shows a comparison of the Raman spectra of the NCD samples prepared at the lowest (1 × 10^14^ cm^−2^) and highest (5 × 10^15^ cm^−2^) implantation fluences and annealed. For the lowest fluence, the spectra (Figure 5a) almost did not change after annealing at different temperatures. In contrast, for the higher fluence (Figure 5b), an increase in D-band intensity was observed, which indicates a more disordered structure with a lower amount of the *sp*^3^ phase (see also Table 3). Consecutive annealing at 800 °C resulted in a further almost 4% decrease of the *sp*^3^ amount (according to the G-band position) in comparison with the as-implanted samples. Similar results were obtained for the implanted single-crystalline diamond published by Kalish, et al. [27]. The amount of *sp*^3^ hybridised amorphous carbon in the layer is comparable with the results published by [33], where they had about 20–30% of *sp*^3^ carbons. The increase of the D-band with increasing annealing temperature is related to an increase in the size of the graphitic crystallites comprising the implanted film [32].

The structure of the single-crystal diamond is characterized by a single sharp peak at 1332 cm^−^^1^ (see Figure 5c). The post-implantation annealing caused the same effect as was observed in NCD samples. The D-band increased after annealing at 800 °C. We can propose that the annealing of both structures, i.e., single- and nano-crystalline diamond, damaged above a certain amorphisation limit (i.e., implanted using higher fluences 1 × 10^15^ and 5 × 10^15^ cm^−2^) does not recover the diamond structure, but rather it increases the size of the existing graphitic crystallites.

**Figure 6:**
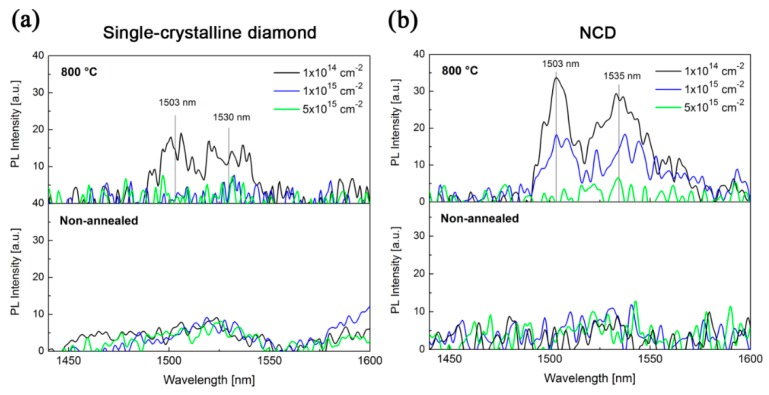
The luminescence spectra of the single-crystalline diamond and NCD samples implanted with various fluences and annealed at 800 °C in vacuum.

Figure 6 shows room-temperature luminescence spectra of the single- and nano-crystalline diamond samples implanted with all three implantation fluences. The as-implanted (and not-annealed) single- and nano-crystalline diamond samples reveal only low-intensity Er-based luminescence. The annealing at 800 °C in vacuum led to an increase of the luminescence intensity for the lowest implantation fluence (1 × 10^14^ cm^−2^). Only for the NCD sample the Er-related PL increased also for the higher fluence of 1 × 10^15^ cm^−2^. In addition, the formation of two distinct luminescence bands was observed for both diamond structures. The main luminescence band is located at around 1530 nm and the second low-intensity luminescence is observed at around 1505 nm. Both luminescence bands are attributed to the transition ^4^I_13/2_ → ^4^I_15/2_ and are a consequence of the fine splitting of the Er (+III) energy levels ^4^I_15/2_ and ^4^I_13/2_ in the crystal field of the diamond structure. Comparing absolute luminescence intensities for both types of substrates, the NCD samples had higher intensity of luminescence than the single-crystalline diamond samples. The samples implanted at 5 × 10^15^ cm^−2^ did not reveal Er-related luminescence at all. 

## 4. Discussion

With the simple presumption that the intensity of luminescence is proportional to the amount of erbium in the measured region (unless there are some other luminescence quenching mechanisms involved such as, e.g., pair-induced clustering luminescence quenching), we may expect that higher fluences of erbium ion implantation will also result in higher luminescence intensity. The ion implantation energy is another of the two key variables for the ion implantation technique (the second one is the ion implantation fluence). The implantation energy is basically ruling the depth in which the majority of atoms will lose their kinetic energy, while the implantation fluence will govern how high the concentration of dopant will be in the implanted structure—basically, higher implantation energy and fluence will result in a deeper dopant penetration and higher dopant concentration, respectively. However, our results do not corroborate this presumption. The fact that no luminescence bands were observed for the highest implantation fluences could be attributed to the luminescence quenching induced by erbium clustering in the surface area due to high local concentration of erbium. Moreover, the luminescence intensity, and mainly the shape of the luminescence spectra, are highly affected by the structure that surrounds the Er ions, and also by whether or not Er (+III) ions are created. According to the RBS/channelling analysis of erbium-implanted single-crystalline diamond, more than 70% of all carbon atoms are out of their original positions and therefore damaged structure can also be expected for NCD films. The same detrimental effect of high Er ion implantation fluences on luminescence was observed for single-crystalline LiNbO_3_ samples implanted at identical conditions [41]. 

For the lower implantation fluences of 1 × 10^14^ and 1 × 10^15^ cm^−2^, we can assume lower damage of the structure and lower total erbium concentration. This was confirmed by our experiments, where for the implantation fluences of 1 × 10^14^ and 1 × 10^15^ cm^−2^ the as-annealed samples showed measurable luminescence, while there was no detectable luminescence in samples implanted with high fluence 5 × 10^15^ cm^−2^. We propose that the main reason why the Er luminescence for the highest implantation fluence of 5 × 10^15^ cm^−2^ (and even 1 × 10^15^ cm^−2^ for the case of single-crystalline samples) cannot be detected is because of the substantial damage of the diamond structure, which leads to the graphitization of the structure. This can be seen from Table 3, where the amounts of *sp*^3^ coordinated carbon in the structure decrease from around 35% to around 25% in the case of higher implantation fluences, while for the lowest implantation fluence, the amount of the *sp*^3^ coordinated carbon is around the same value (33%)—i.e., the samples doped with the lowest implantation fluence are able to recover, while the samples doped with higher implantation fluences (≥1 × 10^15^ cm^−2^) are not able to sufficiently recover.

So far, only a limited number of papers have reported on the luminescence properties of erbium in the diamond-like films. Primarily the groups of Y.M. Foong, et al. and Hui-Lin Hsu, et al. have studied Er (+III) luminescence properties in hydrogenated diamond-like carbon (DLC) films that were doped by erbium ions during the film growth [42,43,44,45]. Erbium concentration in these films was higher than in our case, i.e., of about 3.9 at. %, and the *sp*^2^ hybridisation was predominant in the carbon structure. In their case, only one broad luminescence band centred at 1540 nm was found. This means that the shapes and positions of luminescence bands corresponded well with the electronic transitions of erbium ions and did not differ from our results. A new additional band at 1505 nm was detected in the spectra of annealed samples in our case. This band also corresponds to the electronic transitions of erbium ions and was found for example for the Er:LiNbO_3_ single crystals [16,18]. The luminescence intensities of Er-doped NCD samples could not be compared with the other results due to the different experimental conditions. However, in comparison with the Er-doped single-crystalline samples (e.g., Er:LiNbO_3_, Er:Al_2_O_3_ or Er:ZnO) investigated by our group and measured using the same setup conditions (and using a standard for comparison between measurements), the luminescence intensities of the prepared NCD samples were several orders lower [18,41], indicating that the diamond structure is not as suitable for high Er luminescence as it is for other oxide crystalline materials. 

The beneficial effect of post-implantation annealing on Er luminescence intensity in various matrices has been proven many times. However, no report has been found yet for erbium in a diamond-like matrix. Since diamond has a metastable crystal lattice, there is a critical damage level above which the disrupted crystalline structure cannot be recovered using post-implantation annealing. The diamond will therefore rather transform to a graphite-like structure, i.e., to *sp*^2^ hybridisation. On the other hand, for damage densities below the critical damage level, the diamond structure can be recovered to a very high extent back to *sp*^3^ hybridisation. For the single-crystalline diamond structure, the critical damage level is 10^22^ vacancies·cm^−3^ [46]. Nevertheless, the positive effect of annealing at 800 °C in contrast to the non-annealed samples was proven for the samples implanted with the lowest implantation fluence of 1 × 10^14^ cm^−2^, where a two-fold increase of the luminescence intensity after the annealing was observed. The crucial factor for the recovery and redistribution of erbium ions in the NCD samples is the choice of the annealing temperature. The annealing effect of the temperature of 800 °C is in good agreement with the recrystallization temperature of damaged NCD as reported by Kalish et al. [27]. There are also other ways (apart from annealing) to influence the vicinity of Er ions in a diamond structure and enhance the Er luminescence even further. As we showed recently, one of the more promising ways is a co-doping of Er with some other co-dopants, including the widely known Er sensitizer Yb [47]. 

## 5. Conclusions

Optically active erbium luminescence centres were created in single- and nano-crystalline diamond samples using the ion implantation at 190 keV with fluences up to 5 × 10^15^ ions·cm^−2^ followed by high-temperature annealing in vacuum. The Rutherford Backscattering Spectrometry results showed that Er concentration depth maxima were slightly deeper for NCD films as compared with the single-crystalline samples. The total number of displaced atoms exhibited the concentration maximum in the depth of about 40 nm. The post-implantation annealing was found as a crucial technological step leading to a diamond structure recovery as well as to the enhancement of the Er-based luminescence intensity. Regarding the diamond structure recovery, better results were observed for the annealing in a vacuum than annealing in the Ar atmosphere. For the lowest fluence the post-implantation annealing at 800 °C enhanced the Er luminescence for both substrates (NCD and diamond). Moreover, two distinct luminescence bands at around 1505 and 1535 nm instead of one broad luminescence band were observed, which is beneficial for future applications such as lasers and optical amplifiers. 

## Figures and Tables

**Table 1:** The parameters of the deconvolution fitting process.

Peak No.	Peak Position	Peak Fitting Function	Peak/Band Description
1	≈1150 cm^−1^	Voight	t-Pa = trans-polyacetylene (ω_1_)
2	≈1200–1250 cm^−1^	Gaussian	d_nc_ (VDOS) = diamond crystallites
3	1332 cm^−1^	Lorentzian	d-peak = diamond
4	≈1350 cm^−1^	Voight	D-band (disordered) = *sp*^2^ rings
5	≈1480 cm^−1^	Voight	t-Pa = trans-polyacetylene (ω_3_)
6	≈1580 cm^−1^	Voight	G-band = all *sp*^2^ atoms in both rings and chains

**Table 2:** The Er concentration depth profile parameters.

Ion-Implantation Conditions	Single-Crystalline Diamond *		NCD	
	*R_P_* [nm]	Δ*R_P_* [nm]	*R_P_* [nm]	Δ*R_P_* [nm]
SRIM Er^+^, 190 keV	40	6	-	-
Er^+^, 190 keV, 1.0 × 10^14^ ions/cm^2^	44	9	42	21
Er^+^, 190 keV, 1.0 × 10^15^ ions/cm^2^	41	12	46	14
Er^+^, 190 keV, 5.0 × 10^15^ ions/cm^2^	44	12	50	15

* These values have been published in [29].

**Table 3:** The data evaluated from the Raman spectra deconvolution procedure for the Er-doped NCD samples.

Sample *	Description	I_D_/I_G_	A_D_/A_G_	I_d_/I(d_nc_)	*sp*^3^ [%] (from G-Band Position) **	*sp*^3^ [%] (from A_D_/A_G_ Ratio) **
REF	Non-implanted	1.20	1.82	0.51	35	33
1 × 10^14^ cm^−2^ (I-303)	As-implanted	0.99	1.26	0.53	30	40
	400 °C	1.00	1.26	0.62	30	40
	600 °C	0.81	0.87	0.42	30	45
	800 °C	0.98	1.40	0.89	33	36
1 × 10^15^ cm^−2^ (I-304)	As-implanted	0.85	1.01	0.09	28	43
	400 °C	0.93	1.21	0.03	25	41
	600 °C	1.11	1.90	0.07	24	31
	800 °C	1.11	1.67	0.00	25	34
5 × 10^15^ cm^−2^ (I-305)	As-implanted	0.81	0.90	0.06	30	43
	400 °C	0.93	1.14	0.00	28	41
	600 °C	1.15	2.13	0.00	25	29
	800 °C	1.11	1.74	0.00	26	32

* All samples were measured using an excitation wavelength of 532 nm; ** The amount of *sp*^3^-coordinated carbon atoms in the samples was determined according to the procedure used in [33].
